# Working Memory Capacity and Psychotic-Like Experiences in a General Population Sample of Adolescents and Young Adults

**DOI:** 10.3389/fpsyt.2013.00161

**Published:** 2013-12-03

**Authors:** Tim B. Ziermans

**Affiliations:** ^1^Department of Clinical Child and Adolescent Studies, Leiden University, Leiden, Netherlands; ^2^Department of Neuroscience, Karolinska Institutet, Stockholm Brain Institute, Stockholm, Sweden

**Keywords:** working memory, PLEs, schizotypy, psychosis proneness, CAPE, adolescence, internet

## Abstract

Working memory (WM) impairment is a common feature in individuals with schizophrenia and high-risk for psychosis and a promising target for early intervention strategies. However, it is unclear to what extent WM impairment parallels specific behavioral symptoms along the psychosis continuum. To address this issue, the current study investigated the relation of WM capacity with psychotic-like experiences (PLEs) in a large Swedish population sample (*N* = 1012) of adolescents and young adults (*M* = 24.4 years, range 12–35). WM was assessed with two online computer tasks: a task where participants had to identify and remember the location of an odd shape and a task of remembering and following instructions. PLE scores were derived from a translated symptom questionnaire (Community Assessment of Psychic Experiences), which includes positive, negative, and depressive symptom scales. Positive and negative symptom scales were further subdivided into symptom clusters based on factor analyses. The results showed that low WM capacity was modestly associated with increased reports of bizarre experiences (BE) and depressive symptoms, after controlling for age, gender, and global symptom scores. Interestingly, when analyses were repeated for separate age groups, low WM was exclusively associated with a higher frequency of BE for young adults (20–27 years) and with depressive symptoms for older adults (28–35 years). These findings suggest that specific PLEs can be indicative of reduced WM capacity in early adulthood, which in turn may reflect an increased risk for psychosis and a greater need for targeted intervention. In contrast, during adolescence individual differences in cognitive development may influence the strength of the relationships and thereby mask potential vulnerabilities for psychopathology.

## Introduction

Working memory (WM) refers to the cognitive function to retain and manipulate information over a brief period of time. Intact WM relies heavily on the dopaminergic regulation of neuronal networks, particularly in the prefrontal cortex [e.g., Ref. (1–3)]. Disrupted regulation has commonly been associated with behavioral expressions observed in a variety of neuropsychiatric disorders, including schizophrenia. Since the groundbreaking work of Goldman-Rakic and colleagues on prefrontal lobe function and the cellular mechanisms of WM (4, 5) the scientific community has largely embraced the notion that WM dysfunction constitutes a fundamental neurocognitive impairment pivotal to the pathogenesis of schizophrenia.

Indeed, meta-analytic evidence supports that impairments in WM, as well as in a wide range of other neurocognitive domains, are a common feature among individuals suffering from schizophrenia (6), schizoaffective disorders (7) and, to a lesser extent, young individuals at clinical high-risk for developing a psychotic disorder (8). The latter findings suggest that WM impairments may already be present during the earliest stages of the disorder when the first symptoms are starting to appear. However, most high-risk studies only recruit help-seeking individuals and rely on categorical group comparisons defined by clinical cut-off scores. These strategies are unfit to address whether WM is associated with sub-clinical symptomatology in the population at large as well. Given the recent surge of interest in WM training programs as an intervention aid in early schizophrenia and high-risk populations (9, 10), it is critical to improve our understanding of the putative relationship between WM performance and psychotic-like symptomatology in order to optimize their use and efficacy.

The extended psychosis phenotype refers to the observation that psychotic symptoms or “psychotic-like experiences” (PLEs) exist on a continuum in the general population with clinical schizophrenia on one end and mild, non-clinical schizotypy on the other (11, 12). A recent estimate indicates that the prevalence of PLEs is 7.2% in the general population and for approximately 20% of these individuals the symptoms will persist over time (13). Although PLEs do not necessarily cause distress or affect daily functioning for a majority of individuals, epidemiological studies do indicate that a high intensity of PLEs is associated with an increased risk for psychosis (14–16). Interestingly, prevalence of symptoms may differ across genders and appears to be higher in adolescence than in adulthood (17, 18), a period when the first PLEs typically begin to appear and WM skills are still under maturation (19). Both gender and age may therefore represent moderating variables of the relation between WM and PLEs.

Very few large-scale studies have examined the relationship between WM and PLEs in non-clinical populations, in part due to the time- and resource-consuming aspects of on-site neurocognitive assessments. In addition, available studies have typically focused on one of three classical schizophrenia symptom groups: positive (e.g., hallucinations, delusions), negative [e.g., blunted affect, social withdrawal (SW)], or disorganized (e.g., odd speech and behavior) symptoms. While most findings indicate that reduced WM capacity is associated with increased symptoms, results are mixed for positive and negative symptoms (20–22). This could partly be due to use of different WM measures and clinical instruments, insufficient differentiation of symptoms clusters or sample bias (most studies recruited students). Consequently, it remains unclear whether WM is more commonly associated with specific schizotypal features.

In the current study the relationship between WM capacity and PLEs was further investigated with a fully automated online assessment procedure in a general population sample of Swedish adolescents and young adults. Previous population studies have demonstrated that PLEs do not represent a homogenous entity and can be divided into different subtypes for both positive and negative symptom dimensions (23, 24). These subtypes may convey a different level of risk for psychotic disorders and therefore also exhibit a different interplay with cognitive functions such as WM. It was expected that positive and negative PLEs would be best represented by previously established subdivisions of symptom clusters (18, 24–26) and that clusters associated most with an elevated risk of psychosis [bizarre experiences, persecutory ideas, perceptual abnormalities] (27) would show the strongest negative association with WM capacity.

## Materials and Methods

### Participants

The study was carried out in a sample of 1087 Swedish citizens between 12 and 35 years of age. Participants were recruited via a company specialized in online data collection and population surveys (http://www.norstat.se). Individuals aged 15 years or older provided their informed consent via an online consent form. For individuals younger than 15 years one of their parents provided consent. The study was approved by the Central Ethical Review Board on Research Involving Humans at Karolinska Institutet. The role of the recruitment company was restricted to generating a study sample representative of the Swedish population and it was not involved in the design or execution of the study. Participants were drawn randomly from a voluntarily registered panel of over 100,000 Swedish citizens. A total of 9582 adult individuals received an invitation to participate and an additional 4652 parents of children between 12 and 18 years received an invitation for participation of their child (total response rate = 7.3%). In addition to meeting the age criterion, participants were required to be fluent in Swedish and to have access to a computer with internet connectivity and an operational sound system.

### Instruments and test procedure

#### Psychotic-like experiences

Psychotic-like experiences were assessed with a Swedish version of the Community Assessment of Psychic Experiences (CAPE) (28). This self-report scale measures the lifetime prevalence of positive, negative, and depressive symptoms on both a frequency scale (0 = never to 3 = nearly always) and a distress scale (1 = not distressed to 4 = very distressed). The CAPE questions were translated from English into Swedish and back-translated to increase reliability. Three qualified Swedish researchers with a clinical background carried out the translation. An independent professional translator completed the back-translation, after which a consensus version was drawn up for implementation as an online survey. Frequency scores were transformed to range from 1 to 4 for further analysis and internal consistency of the total CAPE was high (α = 0.91). The translated questionnaire is freely available online at http://cape42.homestead.com/.

#### Working memory

**Figure 1:**
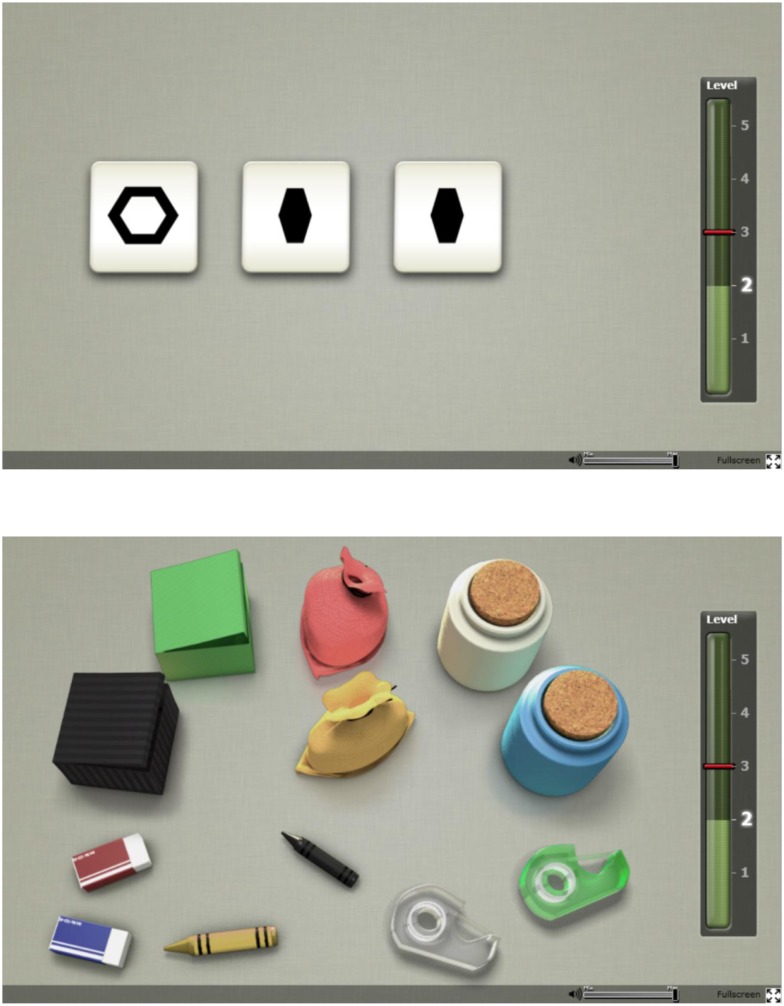
**Screen images of the online working memory tasks: Odd One Out (top) and Following Instructions (bottom)**.

Two tasks of the Cogmed Progress Indicator (CPI) developed by Cogmed, Pearson Assessment, were used to assess WM capacity. The CPI tasks were originally designed to measure WM training improvements with non-trained tasks. The first WM task was the “Odd One Out” [based on a similar task in the Automated Working Memory Assessment (29)] and the second a digital variant of the “Following Instructions” task (30). Figure 1 shows a single frame taken from both tasks.

In the Odd One Out, the participant is prompted to identify which shape out of three is the odd one and then remember its location. The procedure is then repeated with three new shapes after which three empty slots appear. The participant is required to respond by indicating *where* the odd shapes had appeared, in the correct order of appearance. Once the participant has successfully completed the practice trials with two items, the task begins with two items. Two correct trials on each level will lead to progression to the next level where the item load is increased by one. The test is completed when two trials on the same level are incorrect. The final score is calculated based on performance on the highest level achieved where at least one trial was passed, after which 0.3 would be subtracted for each incorrect answer on that level along with 0.15 subtraction for each incorrect answer on levels below the highest level achieved.

The Following Instructions task consists of common classroom items laid out on a table (e.g., eraser, crayon, box) and the task is to follow the verbal instructions given as accurately as possible. The instruction could for instance be “Click on the green eraser, then drag the black crayon to the yellow box,” which would be a trial on span level three (because there are three items to remember what to do with). Practice trials with one and then two items are presented. The task has the same progression, stopping and scoring rules as described for the Odd One Out task above. Subjects exhibiting signs of floor effects (below a cut-off score of 2) were excluded from further data analysis. Finally, scores on both WM tasks were transformed into *z*-scores and an average *z*-score was calculated for each individual as a proxy for WM capacity.

#### Test procedure

After consent was provided, participants received a link to the online assessment together with a unique username and password combination, valid for one assessment only. Participants were strongly advised to complete the assessment in a quiet environment. The order of the WM tasks was always the same [(1) Odd One Out, (2) Following Instructions]. After completion participants were automatically redirected to the web-based CAPE questionnaire. In order to progress each question required an answer, thereby preventing the occurrence of missing values. Participants that completed the assessment received a cinema voucher as a reward. The total duration of the recruitment period was 6 weeks.

### Data analysis

Statistical analyses were performed with IBM SPSS version 20.0. All data were checked for normality, homogeneity, duplicate cases, and outliers. To establish the presence of subtypes of PLEs in the present sample, it was first tested whether the positive symptoms scale contained a similar factor structure as reported in previous studies (18, 25, 26). For this purpose, a confirmatory factor analysis was conducted with IBM Amos 21 and three different models with a four-factor structure were compared. To determine the factor structure of negative symptoms an exploratory factor analysis was preferred, since only one known study (24) has previously reported on this for the CAPE. Frequency scores were entered in a principal component analysis (PCA) with direct oblimin rotation. Three factors had an Eigenvalue ≥1 and the scree plot also indicated a cut-off of three factors. Subsequent parallel analysis with Monte Carlo simulation (31) confirmed that the three-factor structure best represented the data. Kaiser–Meyer–Olkin (KMO) measure of sampling adequacy and Bartlett’s test of sphericity were used to assess whether the factor model was appropriate. KMO was 0.91 and Bartlett’s test significant (*p* < 0.001), which suggested that data properties were excellent for factor analysis (32).

Next, sum scores for all subscales (four positive, three negative) were generated. An additional subscale for depressive symptoms (eight items) was also added for explorative reasons. All subscales were first checked for gender and age differences with non-parametric Mann–Whitney *U* and Kruskal–Wallis tests. For age comparisons participants were divided into three groups with a total age span of 8 years each: “adolescents” (12–19 years), “young adults” (20–27 years), and “adults” (28–35 years). *Post hoc* testing was based on the assumption of unequal sample sizes and variances and checked with Tamhane’s T2 tests. Next, it was assessed whether there were any linear associations between WM and CAPE subscale scores. Spearman’s rho was used for bivariate correlations and the *p*-value was Bonferroni corrected for multiple comparisons (*p* < 0.0063). Multiple hierarchical regression models were used to further investigate the association of WM with symptom scores. For regression analyses *p*-values < 0.05 were considered to be statistically significant.

## Results

### Participants

A total of 1087 assessments were completed. Fifty-one duplicate cases were identified and excluded. Fourteen participants did not meet the age criterion and 10 additional adolescents were excluded after verification that a parent had either partially or completely filled out the CAPE on their behalf. This resulted in 1012 subjects for factor analysis of the CAPE, consisting of 547 female (54%) and 465 male (46%) individuals between 12 and 35 years (*M* = 24.4 years ± 6.6). The geographic distribution of participants was spread over all 21 counties (*län*) of Sweden and central tendency scores (mean, mode, median) for “highest education completed” indicated that a secondary education was the most common and average level of completed education in the sample.

### Confirmatory factor analysis and positive symptom subscales

Model-fitting statistics are provided in Table 1. In sum, the results show that a four-factor solution for the positive symptoms dimension is plausible for our sample. Comparison of the three models indicates that the base model derived from Ref. (18), is the single model that would meet broadly used cut-off criteria for model-fitting indices (33). The model slightly improved when error terms of three item-pairs were correlated. The models based on the factor structure by Yung et al. (25) and Armando et al. (26), required an additional number of correlated error terms to obtain an adequate fit. Based on the comparison, the model of Ref. (18) was adopted in this study to generate sum scores for the subtypes: bizarre experiences (BE), persecutory ideas (PI), perceptual abnormalities (PA), and grandiosity (GR). The factor loadings for both studies are displayed in Table 2.

**Table 1:** **Confirmatory factor analysis: fitting of three different models for the Community Assessment of Psychic Experiences – positive symptoms dimension**.

Models	χ^2^	df	χ^2^/df	CFI	RMSEA	RMSEA interval	AIC
(25) – four-factor model (BE, PI, PA, MT)
Base	729.8	164	4.45	0.85	0.06	0.05–0.06	821.8
+ *r* (q: 2–6, 2–22, 11–13, 15–20, 33–34)	470.9	159	2.96	0.92	0.04	0.04–0.05	573.0
−PI → q41	416.2	141	2.95	0.93	0.04	0.04–0.05	514.2
(18) – four-factor model (BE, PI, PA, GR)
Base	478.1	129	3.71	0.90	0.05	0.05–0.06	562.1
+ *r* (q: 2–6, 2–22, 33–34)	371.1	126	2.95	0.93	0.04	0.04–0.05	461.1
(26) – four-factor model (BE, PI, PA, MT)
Base	735.2	164	4.48	0.85	0.06	0.05–0.06	827.2
+ *r* (q: 2–6, 2–22, 11–13, 33–34)	491.3	160	3.10	0.91	0.05	0.04–0.05	591.3
+ BE → q5, q7 and PA → q31	549.0	163	3.43	0.90	0.05	0.04–0.05	649.0

CFI, comparative fit index; RMSEA, root mean square error of approximation; AIC, akaike information criterion; BE, bizarre experiences; PA, perceptual abnormalities; PI, persecutory ideas; MT, magical thinking; GR, Grandiosity; + ∕− = added/removed; r, correlated error terms; q, question; → = model pathway.

**Table 2:** **Community assessment of psychic experiences: positive symptoms frequency scores and factor analysis**.

	Frequencies (%)		CFA current study				EFA (18)			
	≥Sometimes	Almost always	F1	F2	F3	F4	F1	F2	F3	F4
**BIZARRE EXPERIENCES (BE)**										
26 Do you ever feel as if the thoughts in your head were not your own?	13.3	0.5	0.61				0.68			
28 Have your thoughts ever been so vivid that you were worried other people would hear them?	17.3	0.5	0.58				0.55			
31 Do you ever feel as if you are under the control of some force or power other than yourself?	12.8	0.6	0.58				0.47			
24 Do you ever feel as if the thoughts in your head are being taken away from you?	19.3	0.4	0.51				0.67			
30 Do you ever hear your own thoughts being echoed back to you?	14.9	0.9	0.56				0.49			
17 Do you ever feel as if electrical devices such as computers can influence the way you think?	34.2	1.6	0.47				0.47			
5 Do you ever feel as if things in magazines or on TV were written especially for you?	30.0	0.1	0.33				0.48			
**PERCEPTUAL ABNORMALITIES (PA)**										
42 Do you ever see objects, people or animals that other people can’t see?	6.0	0.6		0.60					−0.76	
34 Do you ever hear voices talking to each other when you were alone?	2.5	0.3		0.56					−0.83	
41 Do you ever feel as if a double has taken the place of a family member, friend or acquaintance?	4.0	0.4		0.48					−0.36	
33 Do you ever hear voices when you were alone?	7.7	0.7		0.47					−0.76	
**PERSECUTORY IDEAS (PI)**										
10 Do you ever feel as if there is a conspiracy against you?	14.7	0.5				0.60				0.49
7 Do you ever feel that you are being persecuted in some way?	16.5	0.2				0.58				0.40
22 Do you ever feel that people look at you oddly because of your appearance?	45.1	1.2				0.54				0.69
6 Do you ever feel as if some people are not what they seem to be?	88.8	1.8				0.39				0.58
2 Do you ever feel as if people seem to drop hints about you or say things with a double meaning?	62.3	3.6				0.32				0.81
**GRANDIOSITY (GR)**										
13 Do you ever feel that you are a very special or unusual person?	69.4	6.7			0.85			0.81		
11 Do you ever feel as if you are destined to be someone very important?	54.2	3.1			0.53			0.82		
**CORRELATION MATRIX**										
F1–BE			1	0.83	0.37	0.75	Correlations not available			
F2–PA				1	0.28	0.65				
F3–GR					1	0.49				
F4–PI						1				

CFA, confirmatory factor analysis; EFA, exploratory factor analysis.

### Exploratory factor analysis and negative symptom subscales

The three-factor solution explained 54% of the variance and all items loaded high (>0.4) on one factor variable and only one item (item 29) had a cross-loading ≥0.25 (Table 3). The first factor was related to avolition (AV) and consisted of seven items, three items loaded on a second factor related to affective flattening (AF) and the last factor consisted of four items related to social withdrawal (SW). Compared to the only available factor structure of CAPE negative symptoms (24) loadings were in high agreement (Table 3). One item (16; “Do you ever feel you have no interest to be with other people?”) loaded highest on SW in the current study as opposed to AV in the previous study and was therefore included in the SW sum score only.

**Table 3:** **Community assessment of psychic experiences: negative symptoms frequency scores and factor analysis**.

	Frequencies (%)		EFA current study			EFA (24)		
	≥Sometimes	Almost always	F1	F2	F3	F1	F2	F3
**AVOLITION (AV)**								
25 Do you ever feel that you are spending all your days doing nothing?	59.7	4.3	0.73			0.59		
36 Do you ever feel that you can never get things done?	88.4	4.8	0.73			0.71		
23 Do you ever feel that your mind is empty?	57.2	1.0	0.69			0.46		
21 Do you ever feel that you are lacking in energy?	91.0	4.7	0.68			0.59		
18 Do you ever feel that you are lacking in motivation to do things?	91.8	4.7	0.66			0.60		
37 Do you ever feel that you have only few hobbies or interests?	59.1	7.9	0.54			0.40		
35 Do you ever feel that you are neglecting your appearance or personal hygiene?	45.5	1.5	0.51			0.62		
**AFFECTIVE FLATTENING (AF)**								
8 Do you ever feel that you experience few or no emotions at important events?	49.3	3.0		0.87			0.70	
27 Do you ever feel that your feelings are lacking in intensity?	40.7	1.8		0.79			0.74	
32 Do you ever feel that your emotions are blunted?	50.4	1.9		0.75			0.72	
**SOCIAL WITHDRAWAL (SW)**								
4 Do you ever feel that you are not much of a talker when you are conversing with other people?	78.6	6.2			0.84			0.71
3 Do you ever feel that you are not a very animated person?	73.5	3.5			0.74			0.69
29 Do you ever feel that you are lacking in spontaneity?	72.1	3.6	0.33		0.50			0.42
16 Do you ever feel you have no interest to be with other people?	76.9	1.7			0.42	0.33		
**CORRELATION MATRIX**								
F1–AV			1	0.36	0.36	1	0.46	0.43
F2–AF				1	0.29		1	0.32
F3–SW					1			1

EFA, exploratory factor analysis.

### Frequency of psychotic-like experiences

Average prevalence of positive symptoms, determined by individuals reporting ≥2 on the frequency scales (=sometimes or more), was relatively high for PI and GR items (respectively 45 and 62%). Fewer individuals reported having experienced BE or PA (respectively 20 and 5%). All prevalence rates substantially decreased as the frequency rate increased (see Table 2). For negative symptoms the average prevalence rate was highest for SW (75%), then AV (70%), and lowest for AF (47%). In concordance with positive symptoms there was a steep decline in prevalence rates of negative symptoms with increased frequency (see Table 3). Depressive symptoms were also relatively common (*M* = 63%; range: 28–93%), with an average of 3% (range: 1–6%) of respondents indicating that the symptoms were almost always present.

### Psychotic-like experiences by gender and age

Average CAPE scores divided by gender and age groups are available in Table 4. There was no overall pattern of gender differences. Female individuals reported higher scores on PI (*U* = 115,642, *p* = 0.011) and depression (*U* = 102,606.5, *p* < 0.001) than males and there was a trend for higher PA in females as well (*p* = 0.053). In contrast, males reported higher frequencies of GR (*U* = 137,215, *p* < 0.026) and AF (*U* = 115,642, *p* = 0.011). Comparisons between age-groups showed that the reported frequency of all symptom scales differed significantly with age (all *p* < 0.05). *Post hoc* testing suggested that positive symptoms were more common in adolescents, while the highest frequency of negative and depressive symptoms was found in the young adult group (Table 4).

**Table 4:** **Community assessment of psychic experiences (CAPE) scores divided by gender and age group**.

Symptom scales	Gender		Statistic	*p*	Age groups			Statistic	*p*
	Female (*n* = 547)	Male (*n* = 465)			Adolescents (12–19 years; *n* = 252)	Young adults (20–27 years; *n* = 429)	Adults (28–35 years; *n* = 331)		
Total CAPE	68.2 ± 12.6	66.1 ± 12.4	*U* = 121,073	0.204	69.0 ± 13.6	68.8 ± 12.4	65.4 ± 11.5	*H* = 17.26	<0.001^a^
Positive	27.0 ± 5.6	26.7 ± 5.4	*U* = 123,427	0.446	29.8 ± 5.9	27.3 ± 5.2	25.8 ± 4.4	*H* = 65.14	<0.001^b^
BE	8.6 ± 2.0	8.8 ± 2.4	*U* = 133,381	0.166	9.5 ± 2.7	8.7 ± 2.1	8.1 ± 1.7	*H* = 81.28	<0.001^b^
PA	4.3 ± 0.7	4.3 ± 1.0	*U* = 121,874	0.053	4.5 ± 1.1	4.2 ± 0.8	4.2 ± 0.7	*H* = 22.73	<0.001^b^
PI	7.9 ± 1.9	7.6 ± 1.8	*U* = 115,642	0.011	8.2 ± 2.3	7.8 ± 1.8	7.4 ± 1.5	*H* = 24.57	<0.001^b^
GR	3.6 ± 1.3	3.8 ± 1.4	*U* = 137,215	0.026	4.0 ± 1.5	3.8 ± 1.4	3.3 ± 1.2	*H* = 38.70	<0.001^a^
Negative	25.9 ± 5.9	26.3 ± 6.3	*U* = 131,296	0.346	25.6 ± 6.4	26.8 ± 6.1	25.5 ± 5.8	*H* = 10.54	0.005^c^
AV	13.5 ± 3.5	13.3 ± 3.6	*U* = 121,441	0.214	13.4 ± 3.6	13.8 ± 3.6	12.9 ± 3.2	*H* = 9.22	0.010^c^
AF	4.4 ± 1.6	5.1 ± 1.9	*U* = 155,342.5	<0.001	4.6 ± 1.8	4.9 ± 1.8	4.7 ± 1.7	*H* = 8.74	0.013^d^
SW	7.9 ± 2.1	7.9 ± 2.1	*U* = 127,192	0.997	7.6 ± 2.3	8.1 ± 2.0	7.9 ± 2.1	*H* = 16.12	<0.001^e^
Depression	14.9 ± 3.8	13.5 ± 3.4	*U* = 102,606.5	<0.001	14.3 ± 3.9	14.7 ± 3.7	14.4 ± 3.4	*H* = 7.08	0.029^c^

BE, bizarre experiences; PA, perceptual abnormalities; PI, paranoid ideations; GR, grandiosity; social withdrawal; affective flattening; avolition; *Post hoc* results (*p* < 0.05).

^a^Adolescents and young adults > adults.

^b^Adolescents > young adults > adults.

^c^Young adults > adults.

^d^Young adults > adolescents (trend: *p* = 0.08).

^e^Young adults > adolescents.

### Working memory capacity

Data of the WM assessment was missing for 16 subjects due to registration errors. Another 92 subjects were excluded based on suspected floor effects on at least one of both WM variables, leaving 904 remaining subjects for WM analysis (*M*_age_ = 24.7 years ± 6.4; 420 males, 484 females). Average performance level was higher for the Odd One Out task (*M* = 5.81, SD = 1.21, range = 2.4–11.0) than for the Following Instructions task (*M* = 4.89, SD = 1.02, range = 2.0–8.4). The Pearson correlation coefficient between tasks was *r* = 0.31, *p* < 0.001. Correlations of both tasks with overall WM capacity (average *z*-score) was *r* = 0.81, *p* < 0.001. When checked, there was no gender difference in WM capacity (*t*_902_ = 0.70, *p* = 0.48).

### Correlations between working memory and psychotic-like experiences

Correlations of CAPE subscale frequency scores with WM capacity were calculated for 904 participants. After correction for multiple comparisons WM showed a negative association with positive symptom subtypes (BE: *r* = −0.13, *p* = 0.00005; PI: *r* = −0.10, *p* = 0.003) and depression (*r* = −0.10, *p* = 0.003). Partial correlations controlled for age and gender gave similar results (BE: *r* = −0.10, *p* = 0.004; PI: *r* = −0.09, *p* = 0.006; depression: *r* = −0.11, *p* = 0.001).

### Multiple regression with working memory and psychotic-like experiences

Subscales correlating with WM capacity (BE, PI, and depression) were entered as dependent variables in hierarchical multiple regression models. Age (*z*-score) and gender were entered first, next WM capacity, and finally the models were checked for interactions with age and gender. For BE the procedure was followed without inclusion of gender terms in the model. An overview of the regression outcomes is provided in Table 5. All models were highly significant (*p* < 0.001) and WM was negatively associated with BE, PI, and depression, regardless of age*_z_* (BE, PI) or gender (PI, depression) effects (all *p* < 0.01). For depression there was an additional interaction effect of age*_z_* × WM (*t* = −2.18, *p* = 0.03). All effect sizes (β) of significant predictors were considered small. To investigate the specificity of the association between WM and the symptom subscales, regression analyses were repeated with total CAPE score entered as an additional covariate. WM remained a significant predictor for BE and depression (both *p* < 0.05) and at trend-level for PI (*p* < 0.06). To further explore the presence of more specific age-effects, the sample was split into the three age-groups described above and regression analyses were repeated. WM significantly predicted BE in the young adult group [β = −0.12, *t*(419) = −2.43, *p* = 0.016] and depression in the adult group [β = −0.11, *t*(321) = −2.04, *p* = 0.046]. There were no significant associations between WM and symptom scores in the adolescent group.

**Table 5:** **Regression models of symptom scores with working memory, age, and sex as predictors**.

Dependent variable		Block 1			Block 2			Block 3		
		*B*	SE *B*	β	*B*	SE *B*	β	*B*	SE *B*	β
Bizarre experiences	(Constant)	8.68	0.07		8.68	0.07		8.69	0.07	
	Age*_z_*	−0.54	0.07	− 0.25**	− 0.52	0.07	− 0.24**	− 0.53	0.07	− 0.25**
	Working memory*_z_*				− 0.25	0.09	− 0.09**	− 0.52	0.09	− 0.09**
	Age*_z_* × working memory*_z_*							− 0.07	0.09	− 0.03
	*R*^2^			0.06			0.07			0.07
	Δ*R*^2^						0.01**			0.01
Persecutory ideas	(Constant)	7.88	0.08		7.88	0.08		7.89	0.08	
	Age*_z_*	−0.33	0.06	− 0.18**	− 0.31	0.06	− 0.17**	− 0.31	0.06	− 0.17**
	Gender	−0.24	0.12	− 0.07*	− 0.25	0.12	− 0.07*	− 0.25	0.12	− 0.07*
	Working memory*_z_*				− 0.20	0.08	− 0.09**	− 0.33	0.10	− 0.15**
	Age*_z_* × working memory*_z_*							0.02	0.08	0.01
	Gender × working memory*_z_*							0.26	0.15	0.08
	*R*^2^			0.04			0.04			0.04
	Δ*R*^2^						0.01**			0.00
Depressive symptoms	(Constant)	14.92	0.16		14.94	0.16		14.94	0.16	
	Age*_z_*	−0.05	0.12	− 0.01	− 0.01	0.12	− 0.00	− 0.06	0.12	− 0.01
	Gender	−1.11	0.24	− 0.15**	− 1.14	0.24	− 0.16**	− 1.09	0.24	− 0.15**
	Working memory*_z_*				− 0.49	0.15	− 0.11**	− 0.62	0.21	− 0.14**
	Age*_z_* × working memory*_z_*							− 0.32	0.15	− 0.07*
	Gender × working memory*_z_*							0.29	0.29	0.05
	*R*^2^			0.04			0.05			0.05
	Δ*R*^2^						0.01**			0.01

SE, Standard Error; β = standardized beta values.

*p < 0.05; **p < 0.01.

## Discussion

Understanding how cognitive functions relate to behavioral symptoms in the general population can provide further insight into underlying mechanisms of emerging psychopathology and lead to identification of early intervention targets. WM represents a cognitive function that is commonly compromised in schizophrenia spectrum disorders and in individuals at-risk for psychosis, though little is known about specific contributions of WM impairments to schizotypal symptomatology. This study aimed to investigate whether WM capacity was related to presence of PLEs in a large population sample of adolescents and young adults. WM was negatively associated with subtypes of positive (BE and persecutory ideas) and depressive symptoms, also after adjusting for age, gender, and global symptom scores (trend-level for persecutory ideas). However, effect sizes were small and when divided into different age groups, WM was exclusively associated with BE in young adults (20–27 years) and with depression in the adult group (28–35 years).

Negative associations between WM and PLEs have previously been reported for clinical (34–36) and non-clinical samples (37, 38), but relatively few studies have assessed these relations for multiple schizotypal dimensions and simultaneously accounted for their shared variance in the analyses. Two studies that did apply this approach found that reduced WM is associated with more positive symptoms, which was confirmed by the current study. Schmidt-Hansen and Honey (20) reported a strong association for three out of four WM parameters derived from an *n*-back task (*N* = 289). Likewise, Matheson and Langdon (21) reported a negative association for performance on a Letter-Number sequencing task (*N* = 97). However, both studies also found evidence for a link between WM and negative symptoms. This discrepancy with the current findings may partially be caused by differences in study sample, as both previous studies were conducted in student populations. Furthermore, all three studies used different WM assessments and symptom questionnaires, which restricts direct comparisons. Here, performance on two computerized WM tasks was averaged to create a global estimate of each individual’s WM capacity. For future studies the use of multiple parameters delineating different aspects of WM function could provide further detail about specific contributions to the underlying associations with PLEs.

Several additional findings in this study require further emphasis. First, the presented data confirm the presence of underlying subtypes of CAPE positive and negative symptom dimensions, corroborating earlier findings in clinical (23) and non-clinical (17, 18, 24–26) study cohorts. This strengthens the idea that the classical schizophrenia dimensions do not represent homogenous entities and can be subdivided into meaningful symptom clusters. Second, associations with low WM capacity complement previous observations of increased distress and decreased global functioning for high intensity of bizarre experiences and persecutory ideas (17, 18). It also supports the notion that high frequencies of these types of positive symptoms may reflect an increased vulnerability for psychosis (27). Third, there were marked age differences in symptom frequency, which also affected the strength of associations with WM capacity. Perhaps most surprising was the absence of significant WM associations for adolescent participants. This suggests that although PLEs are more frequent in adolescents (39) they are not necessarily a marker of decreased cognitive capacity. Speculatively, for some adolescents low WM capacity may simply reflect delayed cognitive maturation instead of underlying psychopathology. Fourth, the finding that depressive symptoms were uniquely associated with WM capacity was somewhat unexpected, although these scores tend to correlate highly with bizarre experiences and persecutory ideas on the CAPE questionnaire (18, 23) and may therefore share some overlap in associated cognitive dispositions. However, the association with WM was strongest in adult individuals, which could suggest an age-related shift in the idiosyncratic nature of WM impairment as a vulnerability marker for different types of psychopathology. Although this finding awaits replication, it underlines the divergent validity of the CAPE depression scale, which can be used independently to explore unique relations with cognitive factors of interest in future studies.

The computerized WM tasks used for this study were originally designed to measure WM training improvements or “transfer effects” on non-trained tasks, based on previous findings (40, 41). Despite ongoing debate regarding the actual extent of transfer effects, there is overall agreement that WM training can enhance performance on more complex cognitive tasks in addition to improved WM capacity (42–45). This apparent display of cognitive plasticity is accompanied by underlying functional brain changes, for example in the (striatal) dopamine system (46–48), dysregulation of which is particularly associated with presence of positive symptoms (49). Furthermore, cognitive remediation strategies that target WM and other cognitive functions have proven successful with regard to cognitive and functional outcome in chronic schizophrenia (50, 51), and bear promise for individuals at-risk for psychosis (52). However, cognitive training can be costly, labor intensive, and its efficacy may vary across individuals. Therefore it would be advantageous to select more narrowly defined participant groups with an increased chance of clinical improvement. Based on the current study results it is tempting to speculate that individuals with high expression of specific positive and depressive symptoms can potentially accomplish the greatest clinical gain from WM training. However, in this study WM only explained a very small proportion of variance in CAPE scores (1%). As such, it is deemed unlikely that stand-alone WM training could directly influence the presence of PLEs for most individuals. Notwithstanding, it is possible that WM training can channel its efficacy in a more indirect manner, e.g., by creating more optimal neurocognitive properties to benefit from other types of treatment. Additional research on actual training interventions is needed to further address this issue.

The current study has multiple methodological strengths and limitations that merit additional commenting. By implementing a fully automated online assessment procedure it was possible to reach a large number of participants within a relatively short time period. Moreover, it allowed for recruitment of a study sample that is considered more or less representative of the general Swedish population between 12 and 35 years. Although the sample may not be completely devoid of any selection bias, it contains, for example, more regional and occupational diversity than community or student populations, which are typically used for this type of large-scale studies. In addition, it has been demonstrated that online assessment of CAPE symptoms is robust against symptom simulation (53) and by requiring individuals to answer each individual question in order to proceed, the problem of dealing with missing data was omitted. Regardless, the assessment could have benefited from adding several items to help detect any malingering participants. Furthermore, the monetary incentive (cinema voucher) may have encouraged participants to register more than once. Although extra precaution was taken to prevent this from happening and data were carefully screened for double entries, it could not be verified completely that all registered participants represented unique individuals. Future studies using a similar approach are advised to apply more rigorous personal identification methods (e.g., via social security number) and to refrain from using a monetary incentive, which would circumstantially further reduce the study costs as well. Finally, even though the online cognitive assessments had considerable logistic advantages over on-site assessments, they did not allow for a fully controlled study environment, which may have influenced the outcome. In general, the adopted online assessment strategy has multiple caveats, though more traditional assessment procedures are by no means less prone to measurement error or sample bias. Furthermore, there are substantial advantages of online recruitment and assessment procedures regarding data completion, project duration, and costs.

To sum up, the current study has provided further evidence for the presence of discernible subtypes of positive and negative PLEs in a large population sample of Swedish adolescents and young adults. There was an inverse relation of WM capacity with presence of positive symptom subtypes (bizarre experiences and persecutory ideas) as well as depressive symptoms, which was moderated by age. However, these relations should be interpreted in light of small effect sizes and marginal impact of WM capacity on symptom variability. Together these findings suggest that specific schizotypal traits may be indicative of reduced WM capacity in different age groups and potentially harbor a greater need for targeted intervention.

## Author Contributions

Tim B. Ziermans conceived the idea and methodology of this study, organized participant recruitment and data processing, conducted the statistical analyses, and wrote the final manuscript.

## Conflict of Interest Statement

The author declares that the research was conducted in the absence of any commercial or financial relationships that could be construed as a potential conflict of interest.
